# Bringing cells to the edge

**DOI:** 10.7554/eLife.83789

**Published:** 2022-11-02

**Authors:** Teng Wang, Lingchong You

**Affiliations:** 1 Department of Biomedical Engineering and the Center for Quantitative Biodesign, Duke University Durham United States

**Keywords:** long-range transport, interfacial mechanics, pattern formation, *Pseudomonas aeruginosa*, *Staphylococcus aureus*, bacteria, Other

## Abstract

A network of open channels allows cells and molecular cargo to travel from the center to the periphery of lab-grown colonies of *Pseudomonas aeruginosa*, helping to eradicate competing species.

**Related research article** Li Y, Liu S, Zhang Y, Seng ZJ, Xu H, Yang L, Wu Y. 2022. Self-organized canals enable long range directed material transport in bacterial communities. *eLife* **11**:e79780. doi: 10.7554/eLife.79780.

Multicellular organisms are, in essence, giant cities of specialized cells. Like the intricate infrastructures that transport people and resources through New York or Amsterdam, vessels and vascular tissues ensure that cells, nutrients and signaling molecules can reach the most remote parts of a plant or an animal. Without these systems, an organism fails to grow and survive.

While bacteria typically live on their own, some can form complex multicellular structures in which cells communicate, coordinate their metabolism and even divide up labor (Tsoi et al., 2018; van Gestel et al., 2015). When cultured on flat surfaces, certain bacterial species create colonies that quickly expand to occupy a large surface area. In fact, these structures become much bigger than what would be possible if newly formed bacteria simply ‘crawled’ from the center of the colony to its outskirts (Warren et al., 2019). For instance, a bacterium within a densely packed group will travel roughly the length of a cell in the time that it takes for the population to double in size (Liu et al., 2021). Many processes have been proposed to aid colony expansion, from mechanical forces to chemically driven bacterial movement (Liu et al., 2021; Sourjik and Wingreen, 2012). Now, in eLife, Yilin Wu, Liang Yang and colleagues – including Ye Li and Shiqi Liu as joint first authors – report a new mechanism that allows cells and molecules to travel long distances at a much faster pace than previously expected (Li et al., 2022).

The team (which is based at Nanyang Technological University, the Chinese University of Hong Kong and the Southern University of Science and Technology) focused on the human pathogen *Pseudomonas aeruginosa*. These opportunistic bacteria form colonies that exhibit exquisite patterns when cultured on a flat agar surface (Luo et al., 2021). Even with the naked eye, Li et al. could observe centimeter-long ‘valleys’ running from the center to the edge of a colony. Under the microscope, the valleys turned out to be a complex network of canals containing fluid flowing towards the colony’s periphery. Several hundred micron wide and 5–10 micron high, these open channels allowed cells to travel thousand times faster than what they could have done on their own (Meacock et al., 2021). Once at the edge, the cells settle and may help the colony to expand.

Both mobile and non-mobile strains of *P. aeruginosa* could create the canals, ruling out a role of cell movement in this process. Instead, Li et al. hypothesized that canal formation was driven by a gradient in surface tension, the force that takes place where the atmosphere and the fluids at the surface of the colony meet. This process would be mediated by rhamnolipids, a type of surfactant molecule produced by *P. aeruginosa* and which decreases surface tension. Indeed, Li et al. found that colonies of rhamnolipid-deficient mutants could not form canals, unless rhamnolipids were injected to artificially establish a surface gradient. Additional experiments showed that rhamnolipids accumulated symmetrically in the center of the colony; it is therefore intriguing why canals only emerge in certain regions.

To explore this question, Li et al. measured flow speeds in colonies before canal formation. This revealed that some regions experienced higher shear rates – meaning that the fluid moved in such a way that it exerted a stronger parallel force onto the cells. The canals developed as fluid flows in the high shear domains carried cells away, a phenomenon known as shear-induced banding (Divoux et al., 2016). A mathematical model accounting for these mechanisms was able to recapture the timing and spatial dynamics of canal formations.

Strikingly, the team found that, in addition to cells, the canals were also transporting outer membrane vesicles (Figure 1). These ‘bag-like’ structures are released by *P. aeruginosa* and often contain compounds essential for bacterial infection, defense or communication (Schwechheimer and Kuehn, 2015). Being able to quickly transport these vesicles across long distances could therefore help *P. aeruginosa* to fight off their competitors. Further experiments showed that colonies of the bacteria *Staphylococcus aureus* quickly died when they encountered *P. aeruginosa* canals, but survived when they were away from them. These results suggest that canal formation can help *P. aeruginosa* establish its population by eradicating competing pathogens.

**Figure 1.**
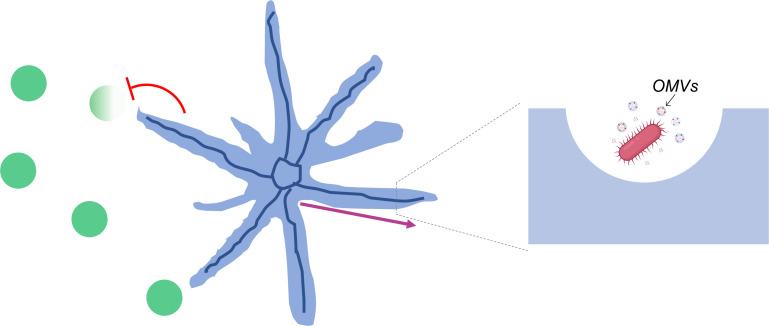
Canals that spontaneously develop in *P*.*aeruginosa* colonies enable the long-range directed transport of materials. Populations of *P. aeruginosa* bacteria (blue) can adopt a branching pattern under certain conditions. Each branch hosts a single self-organized canal (deep blue) which quickly carries cells (insert; pink) and outer membrane vesicles loaded with molecular cargo (insert; OMVs) from the center to the edge of the colony (pink arrow). These canals help *P. aeruginosa* eradicate (red flathead arrow) colonies of competing species (green).

It will be intriguing to explore what other physiological functions these structures may have: could they also help bacterial communities to survive antibiotics, lack of resources and other environmental stresses? In particular, fast transportation could enable cells to explore their space more effectively, potentially making a population more competitive by allowing it to expand quickly when nutrients are scarce (Liu et al., 2021).

This study opens the door to many questions that call for future investigation. In the laboratory, canal formation and fluid flow required specific conditions, such as certain levels of humidity. In addition, the canals observed in wildtype colonies were less stable than those in populations of non-mobile mutants (which were used for most of the experiments); in natural settings, the emergence of the channels could easily be disrupted by bacteria being able to move. It therefore remains to be seen how prevalent canals are in the wild, whether they help *P. aeruginosa* colonize and cause disease in patients, and if they exist in other species.

Even if bacterial canals turn out to be limited in nature, the work of Li et al. opens interesting new avenues for industry. Self-organized bacterial communities can be harnessed to produce valuable chemicals and materials such as biofuels or biodegradable plastics (Kantsler et al., 2020). Being able to modulate how molecules are transported across long distances could allow scientists to better control synthetic communities, for example by loading vesicles with compounds which shape how a colony organizes itself in time and space.
